# Supplementary figures and images for: Early Phase Increase in Serum TIMP-1 in Patients with Acute Encephalopathy with Biphasic Seizures and Late Reduced Diffusion
Source: Children (Basel). 2022 Dec 30;10(1):78. doi: 10.3390/children10010078 (PMC9857315; doi:10.3390/children10010078)

Supplemental Figure S1.

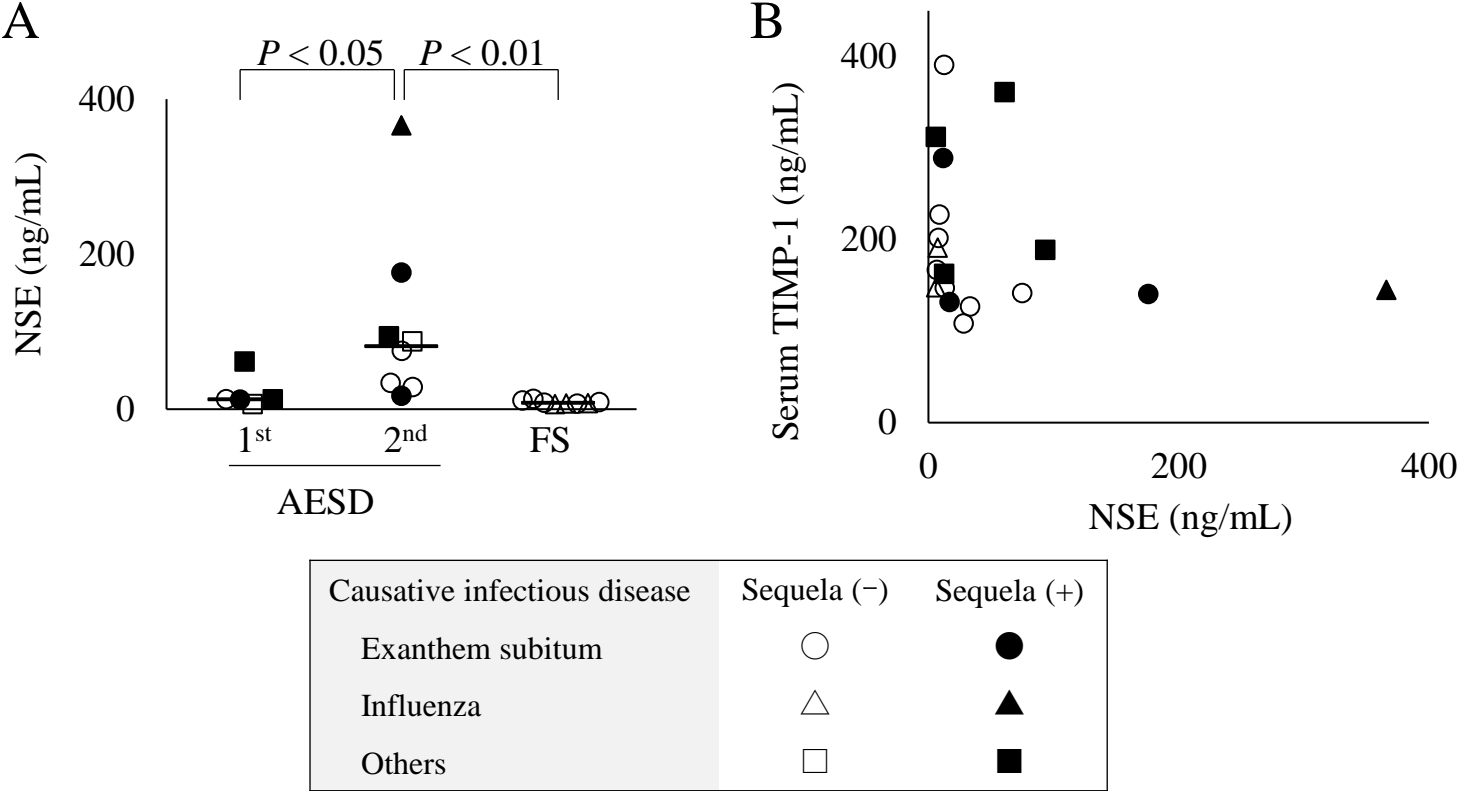

Supplement: Supplementary file 1 [file children-10-00078-s001.zip › children-2045972-supplementary.pdf]
